# Sir John Eric Erichsen

**Published:** 1896-12

**Authors:** 


Sir John Eric Erichsen, F. R. S., LL. D., F. R. C. S., died at Folkestone, England, September 23, 1896, aged seventy-nine years. At the time of his death Mr. Erichsen was Emeritus professor of surgery and consulting surgeon to University Hospital and to many other medical charities. He has been president of the Royal College of Surgeons of England, of the Royal Medical and Chirurgical Society, and of the surgical section of the Great International Medical Congress of 1881. He was surgeon-extraordinary to the Queen, and has been president of University College, London, since 1887. Mr. Erichsen was one of England's best surgeons—and probably England's best teacher of surgery.

